# The impact of an artificial intelligence enhancement program on healthcare providers’ knowledge, attitudes, and workplace flourishing

**DOI:** 10.3389/fpubh.2025.1639333

**Published:** 2025-08-07

**Authors:** Hanaa A. Nofal, Amal E. Mohamed, Noura Almadani, Rasha Mahfouz, Hibah Abdulrahim Bahri, Hossam Tharwat Ali, Dina S. Elrafey

**Affiliations:** ^1^Community, Environmental, and Occupational Medicine Department, Faculty of Medicine, Zagazig University, Zagazig, Egypt; ^2^Community and Psychiatric Mental Health Nursing Department, College of Nursing, Princess Nourah Bint Abdulrahman University, Riyadh, Saudi Arabia; ^3^Medical-Surgical Nursing Department, College of Nursing, Princess Nourah Bint Abdulrahman University, Riyadh, Saudi Arabia; ^4^Qena Faculty of Medicine, South Valley University, Qena, Egypt

**Keywords:** artificial intelligence, healthcare provider, knowledge, attitude, workplace

## Abstract

**Background:**

The integration of AI into healthcare influences healthcare providers’ knowledge, attitudes, and workplace flourishing, grounded in key theoretical frameworks. Social cognitive theory suggests AI-enhanced programs may shape knowledge acquisition and decision-making. The Theory of Planned Behavior helps explain how perceptions of AI affect professional attitudes. Meanwhile, workplace flourishing aligns with positive organizational psychology, emphasizing autonomy and engagement factors potentially impacted by AI adoption. We aimed to examine the impact of artificial intelligence enhancement programs on the knowledge, attitudes, and workplace flourishing of healthcare providers.

**Methods:**

The present study was a quasi-experimental study conducted on healthcare providers at Zagazig University Hospital. The data was gathered using a self-administered three-domain tool, including an artificial intelligence knowledge domain, general attitudes toward artificial intelligence domain, and a workplace flourishing domain.

**Results:**

Regarding the artificial intelligence technologies knowledge, attitude, and flourishing at work scales, post-intervention scores of all domains showed a statistically significant increase compared to pre-intervention, with a percent increase in knowledge score, attitude, and flourishing at work score were 123.14, 74.28, and 10.63%, respectively. Post-intervention attitude score was significantly positively correlated with knowledge score (*p* = 0.001). In addition, age and years of experience were negatively correlated with changes in knowledge and attitude.

**Conclusion:**

Artificial intelligence training is essential for enhancing healthcare providers’ knowledge and alleviating their concerns regarding its integration into healthcare.

**Clinical trial registration:**

Identifier PACTR202403647083094; https://pactr.samrc.ac.za/TrialDisplay.aspx?TrialID=27347

## Introduction

Healthcare systems worldwide face significant challenges in achieving the four primary goals of healthcare: enhancing population health, optimizing patient care experiences, improving caregiver well-being, and reducing escalating healthcare costs. Addressing these interconnected objectives requires strategic reforms, evidence-based interventions, and efficient resource allocation to ensure sustainable, high-quality healthcare delivery (1–3). Aging populations, the increasing prevalence of chronic diseases, and rising healthcare costs pose significant challenges for health systems, necessitating innovation and transformation in care delivery models. Healthcare systems must balance performance by delivering high-quality, effective care while scaling transformation through data-driven insights integrated into patient care. Additionally, the COVID-19 pandemic has further exacerbated disparities in access to care and led to healthcare workforce shortages, underscoring the need for sustainable and equitable healthcare solutions (4). By 2030, the disparity between the supply and demand of the healthcare workforce could widen, reaching nearly 250,000 full-time equivalent positions. Increasing healthcare demands are projected to result in a deficit of 18 million healthcare practitioners, including a shortfall of 5 million physicians, insufficient to meet the healthcare needs of society. This highlights a critical need for proactive workforce planning and sustainable staffing solutions (4, 5).

The incorporation of artificial intelligence (AI) in healthcare is significantly transforming patient care and improving outcomes. AI-driven predictive analytics enhance the efficiency, precision, and cost-effectiveness of disease diagnosis and clinical laboratory assessments, enabling early detection, individualized treatment protocols, and optimized resource allocation. Furthermore, AI supports population health management and the development of evidence-based guidelines by providing real-time, high-fidelity data and improving medication selection. The integration of AI into virtual healthcare services and mental health support has also demonstrated substantial potential in advancing patient outcomes. Nevertheless, mitigating algorithmic biases and addressing limitations in personalization remain essential to ensure equitable, ethical, and effective AI deployment in healthcare (6).

Ensuring the responsible and effective use of AI in healthcare requires a multifaceted approach. Robust cybersecurity frameworks and advanced security protocols must be implemented to protect patient data and safeguard critical healthcare infrastructure. In addition, collaboration among healthcare institutions, AI researchers, and regulatory authorities is essential to develop standardized guidelines for governing AI algorithms and their application in clinical decision-making. Furthermore, sustained investment in research and development is crucial to drive AI innovations that effectively address complex healthcare challenges and improve patient outcomes (7).

“Flourishing” refers to a state of optimal well-being characterized by both eudaimonic well-being (functioning effectively, achieving personal growth, and fulfilling one’s potential) and hedonic well-being (experiencing positive emotions and life satisfaction) (8). According to Diener et al. (9), eight key characteristics of flourishing have been identified: meaning, purpose, healthy relationships, involvement, competence, self-respect, optimism, and social contribution and connections. The PERMA Model was also used to achieve own wellbeing during work placement (positive emotion, engagement, relationships, meaning, accomplishment) (10). These attributes can enhance productivity and well-being among healthcare providers, fostering a more effective and engaged workforce.

Healthcare professionals devote a substantial portion of their time to tasks such as documenting, reviewing, and synthesizing patient information (11, 12). This administrative workload, while essential, is a major source of time inefficiency and has been recognized as a significant factor contributing to professional burnout (13). Burnout, a psychological condition arising from sustained occupational stress (14), has been associated with negative implications for clinician well-being, heightened risk of workforce turnover, and diminished quality and safety of patient care (13, 15, 16). AI comprises a spectrum of sophisticated computational techniques, including natural language processing (NLP), deep learning, intelligent robotics, and context-aware computing (17). These technologies have demonstrated considerable potential in augmenting clinical decision-making and enhancing diagnostic precision. Specifically, AI algorithms are capable of processing and interpreting extensive volumes of medical data, such as radiological images including X-rays, MRIs, and CT scans—thereby supporting more accurate and timely diagnoses.

It is critical to focus on improving the efficiency and effectiveness of the interaction between AI and human intelligence when developing AI systems in healthcare, as AI is designed to augment, not replace, human intelligence. Moreover, AI innovations in healthcare can help unravel the complex nature of care pathways, ensuring that technology complements rather than diminishes the role of healthcare professionals (18). AI will play a fundamental role in the future of medical care and is expected to significantly impact healthcare in the coming years. The integration of AI with telemedicine, genomics, robotics, and 3D printing will advance disease diagnosis and management. Competent healthcare providers undoubtedly have a significant impact on an organization’s success. This study aimed to examine the impact of an AI optimization strategy on healthcare providers’ knowledge, attitudes, and professional practices, to maximize AI-driven benefits in clinical settings.

## Methods

### Study design

This study was a quasi-experimental study conducted on healthcare providers (highly educated) at Zagazig University Hospital from March 2024 to the end of June 2024. It was approved by the Institutional Research Review Board (IRB) of Zagazig College of Medicine (IRB #228/10 March 2024), and this trial was registered on the Pan African Clinical Trial Registry (PACTR202403647083094) at 28/03/2024. The research team explained the study objectives and procedures to participants and assured them that all data would remain confidential. Informed consent was obtained from all participants. All experiments were conducted in strict accordance with relevant ethical guidelines and regulatory standards, ensuring that participants were not exposed to harm or unintended consequences. Furthermore, the study adhered to the ethical principles outlined in the Declaration of Helsinki.

### Study participants

**Figure 1:**
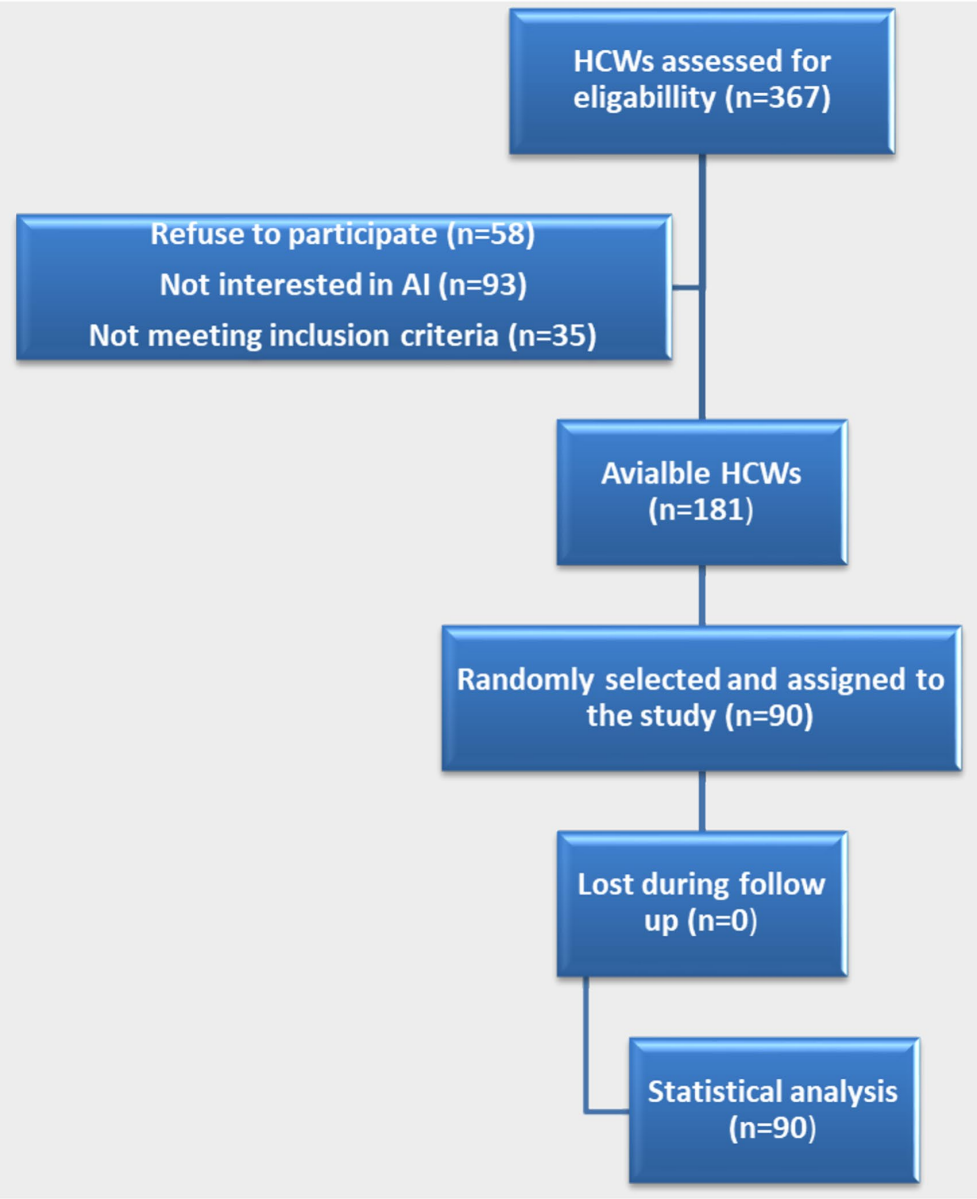
Sampling process of the study participants.

Ninety healthcare providers were selected using a simple random sampling technique. The sample size was calculated using G*Power software version 3.1.9.2, based on an expected small effect size of the intervention program between pre- and post-measurements (d = 0.3), with a 95% confidence interval and 80% power. The required sample size was determined to be 90 (Figure 1).

### Operational design

The study was carried out through three stages. *In the first pre-intervention stage (pre-intervention)*, data were collected using a self-administered three-domain questionnaire assessing knowledge, general attitudes toward AI, and flourishing at work scale. *During the second intervention phase (intervention),* a series of educational sessions were conducted to enhance healthcare providers’ understanding of AI. These sessions covered the concept, significance, and key characteristics of AI, uses, role, principles, and benefits of AI in healthcare, AI strategy development and implementation, and obstacles of AI adoption and potential solutions. Each session commenced with a clear definition of learning objectives. The training program consisted of two sessions per week, each lasting one hour, and lasted for three months. The program included information regarding AI skills, needs, strengths (e.g., diagnosis speed, communication), weaknesses, training needs, and the possibility of personalized training (e.g., virtual practice), hence, how it helps healthcare workers improve. Besides, it was discussed how AI can boost workplace happiness, reduce workload, save time, and increase job satisfaction. *In the third post-intervention phase*, healthcare providers were required to reassess their knowledge and attitudes by completing the same questionnaire assessing knowledge, general attitudes toward AI, and flourishing at work scale. This phase was done to evaluate the effect of the intervention on AI knowledge, attitudes, workplace competencies, and overall well-being among healthcare providers.

### Study tool

The study tool included three main domains in addition to the demographic data (age, marital status, gender, educational level, and years of professional experience). This questionnaire was developed by researchers to assess healthcare providers’ knowledge of AI. Before starting the study, the questionnaire was translated into Arabic and back translated through a specialized bilingual, and afterwards, it was submitted to a board of public medicine experts to judge the tool items for relevance and appropriateness. Besides, each domain has been tested for reliability in prior studies.

*The first domain* was to assess *AI knowledge* based on participants’ prior understanding and post-training evaluation (19–23). This domain includes 35 questions in three formats; True/False (15 questions), Multiple Choice (10 questions), and Matching (10 questions), These questions are grouped into seven domains: Definition of AI (3 questions), Importance and benefits of AI (13 questions), Core components and characteristics (5 questions), Barriers to AI implementation (3 questions), Role of AI and implementation strategies (3 questions), AI principles (5 questions), and AI applications in healthcare (3 questions). The items are shown in the Supplementary File. Each correct answer, regardless of question type, is awarded 1 point. Incorrect answers receive 0 points. Healthcare providers’ AI knowledge levels are categorized as follows: Adequate AI knowledge: ≥ 60%, Inadequate AI knowledge: < 60% (19–24). This domain has been tested for reliability (*α* = 0.79) (19).

*The second domain* was to assess *the general attitudes toward AI* (19–25). This scale consists of 20 items designed to evaluate individuals’ attitudes toward AI. Each item is rated on a 5-point Likert scale: (5 = Strongly Agree, 4 = Agree, 3 = Neutral, 2 = Disagree, 1 = Strongly Disagree). The items are shown in the Supplementary File. Attitude scores are classified based on percentage: Positive attitude toward AI: ≥ 60% and negative attitude toward AI: < 60% (19–26). The questionnaire was tested for reliability (*α* = 0.88) (25).

*The third domain* was on *flourishing at work* (9, 19). The Flourishing at Work Scale measures workplace well-being using eight domains. Each item is valued on a 7-point Likert scale, ranging from 7 = strongly agree and 1 = strongly disagree. The items are shown in the Supplementary File. The cutoff point for interpretation is determined based on the median of the dataset, providing an evidence-based measure of workplace flourishing and overall well-being. The questionnaire was tested for reliability (*α* = 0.87) (9).

### Data management

Data were digitized and statistically analyzed using SPSS version 27.0. Qualitative data were presented as frequencies and relative percentages, while quantitative data were expressed as mean ± standard deviation (SD) or as median and range, depending on data distribution after application of the Kolmogorov–Smirnov normality test. The percent of change was calculated according to the following formula: (“Post score – pre score”/pre score)*100. A paired t-test, Wilcoxon, and McNemar tests were used to compare the results pre- and post-intervention. The Mann–Whitney test, Spearman’s, and Pearson’s correlation were used to find the relation between variables according to data type. *P*-value of <0.05 indicates significant results, and <0.001 indicates highly substantial results.

## Results

The study was conducted on 90 healthcare providers, aged between 24 and 51 years, with a mean age of 34.61 ± 7.61 years. Regarding gender, 57.8% were female, and 51.1% of the participants were from rural areas. The majority were married (84.4%), and most held postgraduate degrees (Master’s or MD). Approximately three-quarters of the participants were physicians, with 50% having worked for five to less than 10 years. Notably, 90% of the participants had no prior training in AI.

**Figure 2:**
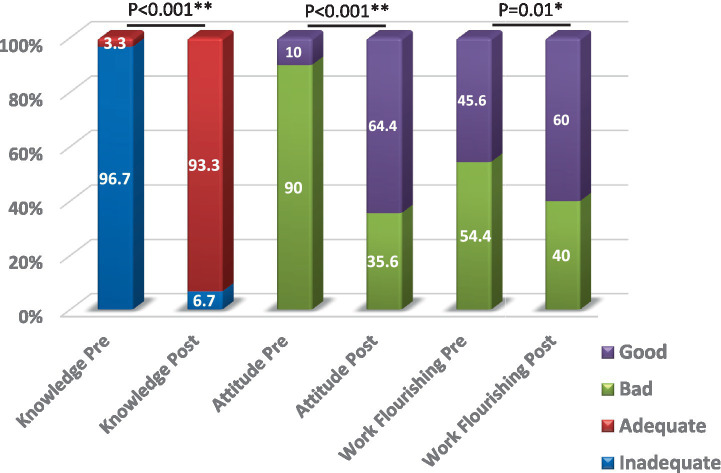
Knowledge and attitudes toward artificial intelligence and work flourishing among the studied HCWs pre & post intervention P: McNemar test, *: significant (*p* < 0.05), **: highly significant (*p* < 0.001).

Table 1 presents the change in the results of the questionnaire domains. There was a statistically significant increase in scores across all domains and the total knowledge score post-intervention compared to pre-intervention, with a 123.14% increase in the overall knowledge score. The highest percent of knowledge improvement was found in principles of AI in the health field (152.13%), and the least change was in the application of AI in the health field (81.82%). Additionally, there was a highly statistically significant improvement in attitude scores post-intervention, with a 74.28% increase. The flourishing score also showed a statistically significant increase in post-intervention, with a 10.63% improvement. Figure 2 illustrates that the frequency of adequate knowledge, positive attitudes, and high work flourishing, all statistically significantly increased post-intervention compared to pre-intervention: adequate knowledge: 3.3 to 93.3%, positive attitude: 10 to 64.4%, and high work flourishing: 45.6 to 60%.

**Table 1:** Artificial intelligence technologies knowledge, general attitudes toward artificial intelligence, and flourishing at work scales pre- and post-intervention among the studied healthcare providers.

Variable	Pre (*n* = 90)	Post (*n* = 90)	Test	*P*	% of change
Definition of AL and how it works	1 (0–2)	3 (1–3)	7.86	<0.001**,†	136.54%
Benefits and importance of AI	5.01 ± 1.8	10.58 ± 1.23	27.67	<0.001**,‡	143.17%
Core components and characteristics of AI	2 (0–5)	4 (1–5)	7.53	<0.001**,†	127.15%
Barriers of using AI in health field	1 (0–3)	3 (1–3)	7.85	<0.001**,†	128.08%
Role and strategies	1 (0–3)	2 (1–3)	7.46	<0.001**,†	91.13%
Principles of AI in health field	1 (0–3)	4 (1–5)	7.74	<0.001**,†	152.31%
Applications of artificial intelligence that can help the nurses	1 (0–2)	3 (1–3)	7.05	<0.001**,†	81.82%
Total knowledge	12.28 ± 2.87	26.34 ± 3.24	39.27	<0.001**,‡	123.14%
Attitudes	36.84 ± 12.24	61.57 ± 8.12	15.13	<0.001**,‡	74.28%
Flourishing at work	35.62 ± 7.52	37.99 ± 6.17	3.27	0.002*,‡	10.63%

Data presented as mean ± SD or median (range), †: paired Wilcoxon test, ‡: paired t-test, *: significant (*P* < 0.05), **: highly significant (*P* < 0.001).

Upon examining the correlation between the different post-intervention scores among the healthcare providers studied, a statistically significant positive correlation was found between post-intervention knowledge and attitude scores (r = 0.33, *p* = 0.001) (Table 2). Table 3 analyzed the relationship between the percentage of change and both sex and occupation. A statistically significant increase in the percentage change in attitude scores was observed among physicians compared to nurses (*p* = 0.01). Table 4 shows the results of the correlation between percentage change and age, education level, and years of experience. Both age and years of experience were significantly correlated with changes in knowledge and attitude scores.

**Table 2:** Correlation between different scales post-intervention among the studied healthcare providers.

Variable	Knowledge (*n* = 149)		Attitude (*n* = 149)	
	r	*P*	r	*P*
Attitude	0.33	0.001*	----	----
Working flourishing	0.03	0.80	0.19	0.07

r: Pearson’s correlation coefficient, *: significant (*p* < 0.05).

**Table 3:** Relation between % of change and sex and occupation.

Variable			*N*	Score		*P*§
				Median	Range	
Knowledge	Sex	Male	38	129.02	28.57–300	0.10
		Female	52	116.67	23.81–200	
	Occupation	Doctor	65	116.77	23.81–300	
		Nurse	25	118.19	42.86–222.22	0.61
Attitude	Sex	Male	38	70.04	12.24–255	0.66
		Female	52	76.03	1.45–270	
	Occupation	Doctor	65	82.35	12.24–270	0.01*
		Nurse	25	48.65	3.03–223.81	
Work flourishing	Sex	Male	38	8.69	1.05–122.22	0.59
		Female	52	12.05	2.92–153.33	
	Occupation	Doctor	65	6.53	2.92–122.22	0.27
		Nurse	25	6.31	1.05–153.33	

§: Mann Whitney test, *: significant (*P* < 0.05).

**Table 4:** Correlation between % of change and age, education, and years of experience among the studied females.

Variable	Change in knowledge (*n* = 90)		Change in attitude (*n* = 90)		Change in flourishing (*n* = 90)	
	r	*P*	r	*P*	r	*P*
Age	−0.26	0.01*	−0.30	0.004*	0.11	0.31
Education	0.14	0.20	0.21	0.05	0.003	0.98
Years of experience	−0.21	0.04*	−0.32	0.002*	0.04	0.72

r: Spearman’s correlation coefficient, *: significant (*P* < 0.05).

## Discussion

As the use of AI in healthcare increases, involving healthcare professionals (HCPs) in AI-enabled technologies will become increasingly crucial. However, HCPs need to be ready for this revolution through AI literacy. Additionally, interventions that improve digital health literacy will enhance the efficiency of the health system and provide opportunities for better health care. This quasi-experimental trial was carried out at Zagazig University hospitals. It aims to examine the ramifications of an AI augmentation paradigm on healthcare practitioners’ proficiencies and occupational flourishing within clinical settings. Assessing health care workers’ knowledge will assist in identifying their educational needs regarding AI integration in clinical settings.

The present study found a knowledge gap that may be due to the need for more training, education, or academic curriculum for undergraduates or postgraduates. The low knowledge reported by the current study can be justified by the fact that 90 % of the healthcare providers studied did not receive any training regarding AI. Also, it is attributed to healthcare students needing to complete training courses in this field. The current study found a statistically significant positive correlation between knowledge and attitude regarding AI. This indicates that the participants’ attitudes are influenced by their knowledge and perception of AI benefits. The study revealed that adequate knowledge, good attitude, and good work flourishing all increased post-intervention compared to pre-intervention. This could be explained that with intervention, the study participants recognize that technology will improve access to information and aid in correct decision making for both clinicians and policy makers. This was consistent with an analysis of an intervention program on nurses only, which indicated a significant magnitude effect of change in knowledge, attitudes of AI technology managerial competencies, and work flourishing (19). Although research indicates ongoing efforts to evaluate the outcomes of AI-related education interventions, there remains a lack of standardized assessment tools, limiting the ability to conduct a comprehensive and consistent evaluation of their effectiveness (27). However, a lack of digital health literacy will remain the main barrier hindering technology adoption.

The present study revealed that adequate knowledge pre-intervention was needed to be higher. This is consistent with a Syrian study and another study in Pakistan (28, 29). They found that medical students and Physicians needed better knowledge of AI and its applications. There is a need that AI to be added to the curricula of medical and nursing schools in these countries, especially with the era of digital transformation. Additionally, a recent Egyptian study on medical students revealed that only 61% of participants had satisfactory knowledge of AI (30).

Regarding attitudes toward AI, the current study found a statistically significant increase in attitude score post compared to pre-intervention, with a percentage increase of 74.28%. These findings are consistent with an Egyptian study that revealed medical students and house officers in Egypt have an overall negative attitude towards the integration of AI technologies in healthcare (31). These results were consistent with a systematic review of primary healthcare physicians who had evidence suggesting that knowledge levels were generally low, with self-seeking learning, diverse attitudes, and concerns about losing jobs and the lack of adoption of new technologies. Besides, the practice experience tends to be positive with AI prior training (32). On the contrary, an American study of PHC physicians revealed that PCPs have largely positive attitudes towards AI. However, these attitudes often depend on the setting of implementation. Primary care providers (PCPs) have expressed concerns regarding integrating AI in primary care, primarily related to people-and-process factors and technological challenges (33). The reported barriers in that review influence the use of digital technologies; however, there are facilitators encouraging the use of technology as useful and easy to use. These findings were consistent with a systematic review that examined healthcare students’ attitudes, knowledge, and skills in AI and revealed that although healthcare students had a positive attitude toward AI in medicine, most healthcare students have low knowledge and limited skills in working with it and need more knowledge and skills (34). Digital and healthcare infrastructure variances might explain significant regional differences in knowledge. The negative attitude and low flourishing of work scores of participants pre-intervention may be due to AI concerns. This contradicts a Korean study suggests that the majority of studied physicians believed that AI would complement their roles in the future and that medical students and doctors prefer the usage of AI in the medical field (35). Moreover, post-intervention, both scores significantly improved, which indicates that better knowledge fosters work flourishing and leads to a promising AI perception. Another Egyptian research study on nurses’ perception of AI revealed a positive association between nurses’ perceptions and attitudes toward AI. Nurses believe artificial Intelligence can improve health care, including patient care and clinical decision-making. Comprehensive training enhances patient outcomes and patient–nurse relationships (36). Another two Egyptian studies revealed that most nurses had reasonable perceptions and positive attitudes toward using AI in healthcare (37, 38).

The present study found that age and years of experience were negatively correlated with changes in knowledge and attitude. This could be explained by the fact that most studied groups had post-graduate degrees (master’s and MD). This is like a study on physician therapists, which revealed that educational qualifications and experiences were substantial predictors of knowledge about AI applications. Moreover, their knowledge of AI applications in rehabilitation was less than their knowledge of AI in general (39). Sabra et al. found that nurses’ attitudes towards AI are mainly predicted through age, qualifications, years of experience, and their social status (37).

The present study revealed a statistically significant increase in flourishing score post compared to pre-intervention, with a percentage increase of 10.63%. This is consistent with a survey of nurses (26). On the contrary, another study (40) stated that AI could impact the well-being of employed workers in different occupations. Workers at higher risk of computerization experience lower stress levels but worse health and lower job satisfaction. The well-being of workers is of significant social importance. They emphasized the harm of technological substitution and the benefits of technological complementarity (40). Finally, a systematic review combined with a survey of physicians stated that despite the increasing application of clinical, most of them lack knowledge and practical experience. Overall, participants have positive but discrete attitudes about AI (41). Despite the varied attitudes around clinical AI, there was a consensus that there should be collaborations between AI and human capabilities, with enhanced AI literacy among HCPs. Innovative undergraduate learning opportunities will enhance their digital literacy and facilitate the adoption of AI technology (42). There is a necessity to solve AI adoption barriers and facilitate all aspects of implementation. The concerns towards AI include people-and-process concerns, e.g., healthcare equity, workflow integration, and other technology-related concerns, e.g., AI accuracy, safety, and potential biases (33). These concerns should be addressed through literacy interventions. Successful integration of AI in healthcare requires regulatory and infrastructure-level interventions concerning institutional ethics and data-sharing agreements. Training in AI should be innovative and effective.

### Limitations

The study was conducted at a single institution (Zagazig University Hospital), which may limit the generalizability of the findings to other healthcare settings with different infrastructures, populations, or resources. Quasi-experimental design with the absence of a control group restricts the ability to attribute observed changes solely to the intervention, as external factors could have influenced the outcomes.

The post-intervention assessment was conducted immediately after the program, limiting insight into the long-term retention of knowledge, sustained attitude changes, or lasting improvements in workplace flourishing. The reliance on self-administered questionnaires may introduce response bias, including social desirability or overestimation of knowledge and attitudes. In addition, the study sample consisted primarily of doctors and healthcare providers with postgraduate degrees, which may not reflect the broader healthcare workforce, including less experienced or differently educated providers.

## Conclusion

The AI intervention program significantly enhanced healthcare providers’ work flourishing, knowledge, and competencies, demonstrating its effectiveness and practical value. These findings highlight the critical need to equip healthcare providers with comprehensive AI training to not only expand their technical understanding but also address apprehension surrounding AI integration in clinical practice. Actively involving healthcare professionals in the implementation and application of AI fosters their sense of competence, increases engagement, and contributes to a more resilient and adaptive workforce. Furthermore, integrating AI technologies into medical education curricula is essential to prepare future healthcare professionals for an evolving digital healthcare landscape.

We strongly recommend that future research explore the long-term impact of AI training programs and support real-world applications through initiatives such as national-level implementation, continuous professional development, and follow-up studies to assess knowledge retention and sustained behavioral change. A strategic, well-supported approach to AI integration will be vital in shaping a more innovative, equitable, and effective healthcare system.

## Data Availability

The raw data supporting the conclusions of this article can be provided upon reasonable request.

## References

[1] Berwick DM Nolan TW Whittington J. The triple aim is care, health and cost. Health Aff. (2008) 27:759–69. doi: 10.1377/hlthaff.27.3.759 18474969

[2] Bodenheimer T Sinsky C. From triple to quadruple aim: care of the patient requires care of the provider. Ann Family Med. (2014) 12:573–6. doi: 10.1370/afm.1713, PMID: 25384822 PMC4226781

[3] Feeley D. The triple aim or the quadruple aim? Four points to help set your strategy. USA: Institute for Healthcare Improvement (2017). 28 p.

[4] Kooli C. COVID-19: challenges and opportunities. Avicenna. (2021) 1. doi: 10.5339/avi.2021.5

[5] Kooli C Al Muftah H. Artificial intelligence in healthcare: a comprehensive review of its ethical concerns. Technol Sustain. (2022) 1:121–31. doi: 10.1108/TECHS-12-2021-0029

[6] Bohr A Memarzadeh K. The rise of artificial intelligence in healthcare applications. Artif Intell Healthc. (2020):25–60. doi: 10.1016/B978-0-12-818438-7.00002-2, PMID: 40690924

[7] Kalis B Collier M Fu R. 10 promising AI applications in health care. Harv Bus Rev. (2018):2–5.

[8] Huppert FA. Flourishing across Europe: application of a new conceptual framework for defining well-being. Soc Indic Res. (2013) 110:837–61. doi: 10.1007/s11205-011-9966-7 23329863 PMC3545194

[9] Diener E Wirtz D Tov W Kim-Prieto C Choi DW Oishi S. New well-being measures: short scales to assess flourishing and positive and negative feelings. Soc Indic Res. (2010) 97:143–56. doi: 10.1007/s11205-009-9493-y

[10] Chisale E Phiri FM. PERMA model and mental health practice. Asian J Pharm Nurs Med Sci. (2022) 10. doi: 10.24203/ajpnms.v10i2.7015

[11] Arndt BG Beasley JW Watkinson MD Temte JL Tuan WJ Sinsky CA. Tethered to the EHR: primary care physician workload assessment using EHR event log data and time-motion observations. Ann Fam Med. (2017) 15:419–26. doi: 10.1370/AFM.2121, PMID: 28893811 PMC5593724

[12] Johnson LG Madandola OO Dos Santos FC Priola KJB Yao Y Macieira TGR. Creating perinatal nursing care plans using ChatGPT a pathway to improve nursing care plans and reduce documentation burden. J Perinat Neonatal Nurs. (2024) 39:10–9. doi: 10.1097/JPN.0000000000000831, PMID: 39491050 PMC11781987

[13] Alumran A Aljuraifani SA Almousa ZA Hariri B Aldossary H Aljuwair M. T he influence of electronic health record use on healthcare providers burnout. Inf Med Unlocked. (2024) 50:101588. doi: 10.1016/J.IMU.2024.101588, PMID: 40690924

[14] Maslach C Leiter MP. Understanding the burnout experience: recent research and its implications for psychiatry. World Psychiatry. (2016) 15:103–11. doi: 10.1002/WPS.20311 27265691 PMC4911781

[15] Kernberg A Gold JA Mohan V. Using ChatGPT-4 to create structured medical notes from audio recordings of physician-patient encounters: comparative study. J Med Internet Res. (2024) 26:e54419. doi: 10.2196/54419, PMID: 38648636 PMC11074889

[16] Gesner E Dykes PC Zhang L Gazarian P. Documentation burden in nursing and its role in clinician burnout syndrome. Appl Clin Inform. (2022) 13:983–90. doi: 10.1055/S-0042-1757157 36261113 PMC9581587

[17] Rashid AB Kausik MAK. AI revolutionizing industries worldwide: a comprehensive overview of its diverse applications. Hybrid Adv. (2024) 7:100277. doi: 10.1016/J.HYBADV.2024.100277

[18] Bajwa J Munir U Nori A Williams B. Artificial intelligence in healthcare: transforming the practice of medicine. Future Healthc J. (2021) 8:e188–94. doi: 10.7861/fhj.2021-0095, PMID: 34286183 PMC8285156

[19] Abdullah Mohamed H Gamal Awad S Elgharib Mohamed Mostafa Eldiasty N ELsaid ELsabahy H. Effect of the artificial intelligence enhancement program on head nurses' managerial competencies and flourishing at work. Egypt J Health Care. (2023) 14:624–45. doi: 10.21608/ejhc.2023.287188

[20] Ongena YP Haan M Yakar D Kwee TC. Patients' views on the implementation of artificial intelligence in radiology: development and validation of a standardized questionnaire. Eur Radiol. (2020) 30:1033–40. doi: 10.1007/s00330-019-06486-0, PMID: 31705254 PMC6957541

[21] Lennartz S Dratsch T Zopfs D Persigehl T Maintz D Große Hokamp N. Use and control of artificial intelligence in patients across the medical workflow: single-center questionnaire study of patient perspectives. J Med Internet Res. (2021) 23:e24221. doi: 10.2196/24221, PMID: 33595451 PMC7929746

[22] Shinners L Aggar C Grace S Smith S. Exploring healthcare professionals’ perceptions of artificial intelligence: validating a questionnaire using the e-Delphi method. Digit Health. (2021) 7:20552076211003433. doi: 10.1177/20552076211003433, PMID: 33815816 PMC7995296

[23] Shimon C Shafat G Dangoor I Ben-Shitrit A. Artificial intelligence enabled preliminary diagnosis for COVID-19 from voice cues and questionnaires. J Acoust Soc Am. (2021) 149:1120–4. doi: 10.1121/10.0003434 33639822 PMC7928231

[24] Hussein Mohamed S Abed El-Rahman Mohamed M Farouk Mahmoud S HessienYousef HE. 'The effect of educational program on nurses’ knowledge and attitude regarding artificial intelligence', Egyptian journal of. Health Care. (2023) 14:1110–28. doi: 10.21608/ejhc.2023.312617

[25] Schepman A Rodway P. Initial validation of the general attitudes towards artificial intelligence scale. Comput Hum Behav Rep. (2020) 1:100014. doi: 10.1016/j.chbr.2020.100014, PMID: 34235291 PMC7231759

[26] Elsayed WA Sleem WF. Nurse managers’ perception and attitudes toward using artificial intelligence technology in health settings. Assiut Sci Nurs J. (2021) 9:182–92. doi: 10.21608/asnj.2021.72740.1159

[27] Charow R Jeyakumar T Younus S Dolatabadi E Salhia M al-Mouaswas D. Artificial intelligence education programs for health care professionals: scoping review. JMIR Med Educ. (2021) 7:e31043. doi: 10.2196/31043, PMID: 34898458 PMC8713099

[28] Swed S Alibrahim H Elkalagi NKH Nasif MN Rais MA Nashwan AJ. Knowledge, attitude, and practice of artificial intelligence among doctors and medical students in Syria: a cross-sectional online survey. Front Artif Intell. (2022) 5:1011524. doi: 10.3389/frai.2022.1011524, PMID: 36248622 PMC9558737

[29] Ahmed Z Bhinder KK Tariq A Tahir MJ Mehmood Q Tabassum MS. Knowledge, attitude, and practice of artificial intelligence among doctors and medical students in Pakistan: a cross-sectional online survey. Ann Med Surg (Lond). (2022) 76:103493. doi: 10.1016/j.amsu.2022.103493, PMID: 35308436 PMC8928127

[30] Ghanem OA Hagag AM Kormod ME el-Refaay MA Khedr AM Abozaid OM. Medical students’ knowledge, attitudes, and practices toward generative artificial intelligence in Egypt 2024: a cross-sectional study. BMC Med Educ. (2025) 25:1–10. doi: 10.1186/s12909-025-07329-x, PMID: 40437443 PMC12117742

[31] Allam RM Abdelfatah D Khalil MIM Elsaieed MM el Desouky ED. Medical students and house officers’ perception, attitude and potential barriers towards artificial intelligence in Egypt, cross sectional survey. BMC Med Educ. (2024) 24:1244. doi: 10.1186/s12909-024-06201-8, PMID: 39482613 PMC11529482

[32] Abdulazeem H Meckawy R Schwarz S Novillo-Ortiz D Klug SJ. (2024). Knowledge, attitude, and practice of primary care physicians toward clinical artificial intelligence applications: a systematic review and meta-analysis. Available online at: https://ssrn.com/abstract=4916043 (Accessed February 11, 2025). 10.1016/j.ijmedinf.2025.105945 40286705

[33] Allen MR Webb S Mandvi A Frieden M Tai-Seale M Kallenberg G. Navigating the doctor-patient-AI relationship—a mixed-methods study of physician attitudes toward artificial intelligence in primary care. BMC Prim Care. (2024) 25:42. doi: 10.1186/s12875-024-02282-y, PMID: 38281026 PMC10821550

[34] Mousavi Baigi SF Sarbaz M Ghaddaripouri K Ghaddaripouri M Mousavi AS Kimiafar K. Attitudes, knowledge, and skills towards artificial intelligence among healthcare students: A systematic review. Health Sci Rep. (2023) 6:e1138. doi: 10.1002/hsr2.1138, PMID: 36923372 PMC10009305

[35] Oh S Kim JH Choi SW Lee HJ Hong J Kwon SH. Physician confidence in artificial intelligence: an online Mobile survey. J Med Internet Res. (2019) 21:e12422. doi: 10.2196/12422, PMID: 30907742 PMC6452288

[36] Ahmed AbdelhakamAhmed E Mohamed Sayed Osman Y Farghaly Ali Mohamed A. Artificial intelligence and the future of health care: is it threatening the existence of nursing? Nurses' perception and attitude. Egypt. J Health Care. (2024) 15:1101–14. doi: 10.21608/ejhc.2024.363907

[37] Sabra HE Abd Elaal HK Sobhy KM Bakr MM. Utilization of artificial intelligence in health care: nurses’ perspectives and attitudes. Menoufia Nurs J. (2023) 8:253–68. doi: 10.21608/menj.2023.297411

[38] Abdelkareem SA Bakri MH Ahmed NA. Perception and attitudes of critical care nurses regarding artificial intelligence at intensive care unit. Assiut Sci Nurs J. (2024) 12:163–71. doi: 10.21608/asnj.2024.268448.1782

[39] Alsobhi M Khan F Chevidikunnan MF Basuodan R Shawli L Neamatallah Z. Physical therapists’ knowledge and attitudes regarding artificial intelligence applications in health care and rehabilitation: cross-sectional study. J Med Internet Res. (2022) 24:e39565. doi: 10.2196/39565, PMID: 36264614 PMC9634519

[40] Nazareno L Schiff DS. The impact of automation and artificial intelligence on worker well-being. Technol Soc. (2021) 67:101679. doi: 10.1016/j.techsoc.2021.101679

[41] Chen M Zhang B Cai Z Seery S Gonzalez MJ Ali NM. Acceptance of clinical artificial intelligence among physicians and medical students: a systematic review with cross-sectional survey. Front Med. (2022) 9:990604. doi: 10.3389/fmed.2022.990604, PMID: 36117979 PMC9472134

[42] Abou Hashish EA Alnajjar H. Digital proficiency: assessing knowledge, attitudes, and skills in digital transformation, health literacy, and artificial intelligence among university nursing students. BMC Med Educ. (2024) 24:508. doi: 10.1186/s12909-024-05482-3, PMID: 38715005 PMC11077799

