# Supplementary material for: The impact of an artificial intelligence enhancement program on healthcare providers’ knowledge, attitudes, and workplace flourishing
Source: Front Public Health. 2025 Aug 7;13:1639333. doi: 10.3389/fpubh.2025.1639333 (PMC12367703; doi:10.3389/fpubh.2025.1639333)
Supplement: Supplementary file 1 [file Data_Sheet_1.PDF]

# The impact of an artificial intelligence enhancement program on healthcare providers’ knowledge, attitudes, and workplace flourishing

*Hanaa A. Nofal, Amal E. Mohamed,  Noura Almadani, Rasha Mahfouz, Hibah Abdulrahim Bahri, Hossam Tharwat Ali, Dina S. Elrafey*

## SUPPLEMENTARY FILE

## STUDY QUESTIONNAIRE

# Knowledge Domain

## Domains Breakdown:

| Domain | No. of Questions | Type (Suggested) |
|---|---|---|
| Definition of AI | 3 | 2 T/F, 1 MCQ |
| Importance & Benefits | 13 | 5 T/F, 4 MCQ, 4 Matching |
| Core Components & Characteristics | 5 | 2 T/F, 2 MCQ, 1 Matching |
| Barriers | 3 | 2 T/F, 1 MCQ |
| Role | 3 | 2 T/F, 1 Matching |
| Principles | 3 | 2 T/F, 1 MCQ |
| Applications in Healthcare | 3 | 1 T/F, 1 MCQ, 1 Matching |

## True / False Questions (15 Questions):

1. AI is a field of computer science focused on creating systems capable of human-like decision-making. (True / False)

2. AI always replaces human workers completely in healthcare settings. (True / False)

3. AI can help reduce the time needed to diagnose diseases. (True / False)

4. AI helps improve healthcare efficiency. (True / False)

5. AI enhances patient safety. (True / False)

6. AI reduces healthcare costs. (True / False)

7. AI is capable of learning from large datasets. (True / False)

8. AI can predict health trends. (True / False)

9. AI has no role in improving clinical decision-making. (True / False)

10. AI cannot handle routine administrative healthcare tasks. (True / False)

11. AI assists in personalizing patient care. (True / False)

12. AI systems never make mistakes. (True / False)

13. AI requires continuous updates and monitoring. (True / False)

14. AI supports evidence-based practice. (True / False)

15. AI in healthcare is guided by ethical considerations. (True / False)

## Multiple Choice Questions (10 Questions):

1. What is the primary aim of AI in healthcare?

- A. Replace doctors
- B. Improve efficiency and support decisions
- C. Increase costs
- D. None of the above

2. Which of the following is a core component of AI?

- A. Imagination
- B. Data processing algorithms
- C. Human emotions
- D. Physical strength

3. AI can help healthcare through:

- A. Improving diagnosis
- B. Wasting time
- C. Reducing efficiency
- D. Increasing workload

4. A key benefit of AI is:

- A. Delays in care
- B. Enhanced patient outcomes
- C. Increased errors
- D. Higher costs

5. A barrier to AI implementation is:

- A. Lack of data
- B. Privacy concerns
- C. Both A and B
- D. None of the above

6. AI helps in which area?

- A. Decision making
- B. Clinical procedures
- C. Education
- D. All of the above

7. One principle of AI in healthcare is:

- A. Transparency
- B. Chaos
- C. Bias
- D. Ignorance

8. AI applications in healthcare include:

- A. Patient monitoring
- B. Data analysis
- C. Diagnostics
- D. All of the above

9. One common misconception about AI is:

- A. AI is always 100% accurate
- B. AI learns from data
- C. AI helps reduce costs
- D. AI improves care

10. What is a role of AI in nursing?

- A. Providing direct care independently
- B. Assisting decision making
- C. Eliminating nurses
- D. Making policies

## Matching Questions (10 Questions):

Match the items in Column A with the appropriate answer in Column B.

| Column A | Column B |
|---|---|
| AI in diagnosis | Reduces errors |
| Machine Learning | Analyzing large data sets |
| Robotics in nursing | Performing repetitive tasks |
| AI in healthcare | Improves efficiency |
| AI data analysis | Predicts health trends |
| AI-based systems | Support decision-making |
| Healthcare AI | Enhances patient safety |
| AI algorithms | Learn from data |
| AI in administration | Automates routine tasks |
| AI technology | Requires ethical guidelines |

# Attitudes Domain

**Instructions**:

Below are statements with which you may agree or disagree. Using the 1–7 scale below, indicate your agreement with each item by placing the appropriate number on the line preceding that item.

1 = Strongly disagree
2 = Disagree
3 = Neutral
4= Agree
5 = Strongly agree

**Items:**

1. I am interested in using AI systems manually in my daily life.

2. There are many beneficial applications of Artificial Intelligence.

3. Artificial Intelligence is exciting.

4. Technical Artificial Intelligence can provide new economic opportunities.

5. I would like to use Artificial Intelligence in my own job.

6. An artificially intelligent agent would be better than an employee in many routine jobs.

7. I am impressed by what Artificial Intelligence can do.

8. AI can have positive effects on nurses' well-being.

9. Artificially intelligent systems can help Nurses feel happier.

10. Artificially intelligent systems can perform better than humans.

11. A lot of organizations will benefit from a future full of AI.

12. For routine transactions, I would rather interact with an artificially intelligent system than with a human.

13. I think Artificial Intelligence is dangerous.

14. Organizations use Artificial Intelligence unethically.

15. I find Artificial Intelligence sinister.

16. Artificial Intelligence is used to spy on Nurses.

17. I shudder with alarm when I think of the future uses of artificial intelligence.

18. AI may control nurses.

19. I think artificially intelligent systems make many errors.

20. Nurses like me will suffer if AI is used more and further.

# The Flourishing Domain

**<u>Instructions</u>**:
Below are statements with which you may agree or disagree. Using the 1–7 scale below, indicate your agreement with each item by placing the appropriate number on the line preceding that item.

1 = Strongly disagree
2 = Disagree
3 = Slightly disagree
4 = Neither agree nor disagree
5 = Slightly agree
6 = Agree
7 = Strongly agree

**<u>Items:</u>**

1. I lead a purposeful and meaningful life.

2. My social relationships are supportive and rewarding.

3. I am engaged and interested in my daily activities.

4. I actively contribute to the happiness and well-being of others.

5. I am competent and capable in the activities that are important to me.

6. I am a good person and live a good life.

7. I am optimistic about my future.

8. People respect me.
